# Editorial: Molecular profiles of tunneling nanotubes (TNTs) in human diseases-from 2D cultures to complex tissue

**DOI:** 10.3389/fcell.2024.1461453

**Published:** 2024-08-14

**Authors:** Nataša Resnik, Guénaëlle Levallet, Mariella Errede, Francesca Re, Daniela Virgintino

**Affiliations:** ^1^ Faculty of Medicine, Institute of Cell Biology, University of Ljubljana, Ljubljana, Slovenia; ^2^ University of Caen Normandy, National Center for Scientific Research, Normandy University, Unit of Imaging and Therapeutic Strategies for Cancers and Cerebral Tissues (ISTCT)-UMR6030, GIP CYCERON, Caen, France; ^3^ Department of Pathology, Caen University Hospital, Caen, France; ^4^ Department of Translational Biomedicine and Neuroscience (DiBraiN), Unit of Human Anatomy and Histology, University of Bari School of Medicine, Bari, Italy; ^5^ School of Medicine and Surgery, University of Milano-Bicocca, Monza, Italy

**Keywords:** cancer, in vitro models, glioblastoma, tumour, tunneling nanotubes

## 1 Introduction

Tunneling nanotubes (TNTs) are a type of intercellular communication that has been growing in importance since their discovery by Rustom and colleagues 20 years ago (Rustom et al., 2004). TNTs are tubular membrane nanostructures that facilitate direct communication between cells over distances of 10–200 µm. TNTs can range in diameter from 5 to 1,000 nm. They allow for the transfer of various intracellular components, including organelles, proteins, genetic material, and vesicles, between connected cells. TNTs have been observed in various cell types and play important roles in both physiological and pathological conditions, being implicated in various human diseases (Dubois et al., 2020; Khattar et al., 2022). In tumors, TNTs allow the transfer of oncogenic molecules, such as proteins and genetic material, between cancer cells, contributing to tumor growth, metastasis, and resistance to therapy. They can also facilitate the exchange of signaling molecules between cancer cells and non-tumoral cells in the tumor microenvironment, influencing tumor progression (Sisakht et al., 2023). TNTs also contribute to non-tumoral disease progression and the propagation of pathology, notably throughout the brain by the spread of α-synuclein mis-folds and aggregates implicated in neurodegenerative Parkinson’s disease (Chakraborty et al., 2023). Certain viruses, including HIV, herpes simplex virus, and respiratory syncytial virus, exploit TNTs for intercellular transfer and dissemination within host tissues, acting as conduits for viral particles to move between cells and aiding in viral spread and evasion of the immune system (Okafo et al., 2017; Wang et al., 2023). A crucial role of TNTs during human brain vascularization and tumoral neo-angiogenesis has also been suggested (Errede et al., 2018), as well as their implication in cardiovascular diseases such as atherosclerosis and cardiac ischemia-reperfusion injury (Lakota et al., 2011; Girolamo et al., 2023). These unique insights into the structure and function of TNTs have been the result of *in vitro* and *in vivo* models.

## 2 Commentary on the articles of the Research Topic

The current Research Topic features a collection of four manuscripts, consisting of a mini-review and three research articles. The manuscripts present data ranging from mechanical and structural features of TNTs to their functional role in human diseases and describe a membrane reshaping proteins such as septins. Simone et al. investigated the role of TNTs in glioblastoma (GBM), showing that glial fibrillary acid protein (GFAP), a representative protein of intermediate filaments in glial cells, is a key structural and functional protein of TNTs formed between distant human-derived GBM cells. Moreover, GFAP interacts with mitochondria during the intercellular transfer mediated by TNTs. This event could be involved in the well-known apoptotic escape mechanism that occurs in GBM cells. Given that the formation of TNTs involved the cytoskeleton rearrangements, a potential supportive role could be played by dynamic protein assemblies like septins, regarded as the fourth element of the cytoskeleton. The effect of septins on the membranes is either through direct interactions or through their association with other cytoskeletal elements, i.e., actin filaments and microtubules, or even through the endocytic-sorting complex required for transport complex (ESCRT). Benoit et al. discussed septins in detail. Considering that the formation of TNTs occurs in a complex microenvironment, where not only diseased but also healthy cells are present, Resnik et al. established a novel urothelial cancer-normal co-culture model that is useful to investigate the role of TNTs in cancer progression and recurrence, with the ultimate aim of developing TNT-based cancer therapies. Similar to the discovery of the intermediate filament protein GFAP in TNTs by Simone et al.; Resnik et al. found the intermediate filament protein cytokeratin 7 as a component of TNTs in normal and cancer urothelial cells. The novelty was the identification of the tri-cytoskeletal composition of the TNTs. In the context of the rather fragile nature of TNTs, it is important to emphasize that TNTs are dynamic and physically elastic structures, supported by the work of Resnik et al.; Li et al. In this context, Li et al. have established methods useful for evaluating the mechanical properties of TNTs. This is of particular importance for the development of new therapeutic strategies based on the destruction or exploitation of TNTs.

## 3 The future of TNT research: open questions and challenges

Further research is needed to fully understand the role of TNTs in diseases and to explore their potential as therapeutic targets or diagnostic markers. One possible strategy that can be pursued is to inhibit or disrupt TNT formation using inhibitors of actin polymerization. On the other hand, a promising alternative could be the exploitation of intercellular communication in TNTs. Indeed, TNTs facilitate the transfer of drugs and drug delivery systems (i.e., nanoparticles) between connected cells (Formicola et al., 2019). Next, the fundamental question regarding identification markers for TNTs needs to be addressed. Two decades of TNT research have shown that the molecular, structural, and biophysical identity of TNTs is very diverse and dependent on the cell environment, making it difficult to find a specific marker to unambiguously identify TNTs from other cellular structures such as filopodia and cytonemes. *In vivo*, the identification of TNTs is particularly challenging due to the complexity of the tissue. High-resolution microscopy techniques such as electron microscopy and super-resolution microscopy are required, but these techniques have limitations due to the fragile and transient nature of TNTs and can be technically challenging. In addition to microscopy, interdisciplinary approaches integrating bioinformatics and computational modelling can help predict the behavior of TNTs and their role in cellular networks. CRISPR/Cas9 and other gene-editing technologies can be used to manipulate genes involved in TNT formation and function, potentially providing future insights into their biological roles.

## 4 Conclusion

Supported by recent studies that have clarified many of the biological, biomechanical, and pharmacological aspects of TNTs, these studies further extend the knowledge of these cellular nanostructures through original *in vitro* and *ex vivo* studies, opening up new fields of analysis on the mechanistic dynamics and multiple roles of TNTs. The combination of *in vitro*, *ex vivo*, and *in vivo* models, together with advanced imaging and molecular techniques and interdisciplinary approaches, is essential for a comprehensive understanding of TNTs. Each model offers unique advantages that, when integrated, can provide a detailed and clinically relevant picture of the role of TNTs in health and disease.
